# Prevalence of antibodies against *Ehrlichia* spp. and *Orientia tsutsugamushi* in small mammals around harbors in Taiwan

**DOI:** 10.1186/s13071-016-1318-7

**Published:** 2016-01-27

**Authors:** Kun-Hsien Tsai, Shu-Feng Chang, Tsai-Ying Yen, Wei-Liang Shih, Wan-Jen Chen, Hsi-Chieh Wang, Xue-Jie Yu, Tzai-Hung Wen, Wen-Jer Wu, Pei-Yun Shu

**Affiliations:** Institute of Environmental Health, College of Public Health, National Taiwan University, No. 17, Xu-Zhou Road, Taipei, 10055 Taiwan; Department of Public Health, College of Public Health, National Taiwan University, No. 17, Xu-Zhou Road, Taipei, 10055 Taiwan; Center for Research, Diagnostics and Vaccine Development, Centers for Disease Control, Ministry of Health and Welfare, No.161, Kun-Yang Street, Taipei, 11561 Taiwan; Institute of Epidemiology and Preventive Medicine, College of Public Health, National Taiwan University, No. 17, Xu-Zhou Road, Taipei, 10055 Taiwan; Infectious Diseases Research and Education Center, Ministry of Health and Welfare and National Taiwan University, No. 17, Xu-Zhou Road, Taipei, 10055 Taiwan; Departmentof Pathology, University of Texas Medical Branch, Galveston, TX USA; Department of Geography, National Taiwan University, No. 1, Sec. 4, Roosevelt Road, Taipei, 10617 Taiwan; Department of Entomology, College of Bioresources and Agriculture, National Taiwan University, No. 1, Sec. 4, Roosevelt Road, Taipei, 10617 Taiwan

**Keywords:** *Ehrlichia chaffeensis*, *Orientia tsutsugamushi*, Small mammals, Taiwan

## Abstract

**Background:**

Tick-borne ehrlichiosis and mite-borne scrub typhus represent important emerging zoonotic rickettsial diseases. Although scrub typhus has been recognized by the Taiwanese public health system, information on ehrlichial infections is scarce in Taiwan. In this study, the risk of spread of ectoparasites on rodents through aerial and marine transportation was assessed in international and domestic harbors. Here, we report the first systematic surveillance of seroprevalence against *Ehrlichia* spp. in small mammals on the main island of Taiwan.

**Methods:**

In total, 1648 small mammals were trapped from 8 international ports, 18 domestic fishing harbors, and 7 local public health centers around Taiwan from November 2004 to December 2008. Sera were analyzed using indirect immunofluorescence assays to detect IgG antibodies against *Ehrlichia chaffeensis* and *Orientia tsutsugamushi*. A serum titer of ≧1:80 was considered positive.

**Results:**

Antibodies against *Ehrlichia* spp. and *O. tsutsugamushi* were detected in 3.28 % and 4.92 % of small mammals active around harbors, respectively. The seropositive rate against *Ehrlichia* was higher in northern Taiwan from 2005 to 2008. However, *O. tsutsugamushi* infections increased in southern Taiwan during this period. The serological evidence of ehrlichial and *O. tsutsugamushi* infections in all international ports were included in the study. No significant differences were found among the seropositive rates of *Ehrlichia* spp. and *O. tsutsugamushi* in small mammals trapped between international and local harbors.

**Conclusions:**

The overall prevalence of *Ehrlichia* spp. and *O. tsutsugamushi* infections in small mammals active around harbors was 3.28 % and 4.92 %, respectively. The results provided serological evidence supporting the potential risks of transporting pathogens through air and maritime traffic. This study highlights serious issues of the emergence and spread of rickettsial diseases in Taiwan. The incidence of human ehrlichiosis requires further investigation.

## Background

Rickettsial infections are caused by several genera of obligate intracellular Gram-negative bacteria with life cycles that involve vertebrate and invertebrate hosts. Different types of rickettsiosis are widely distributed worldwide, but the vector species and associated human illnesses are distinct depending on geographical locations. Rickettsioses are classically divided into the scrub typhus group, which is transmitted by mites, the spotted fever group, which is transmitted by ticks or mites, and the typhus group, which is primarily transmitted by lice or fleas. In Taiwan, notifiable rickettsial diseases include scrub typhus, epidemic typhus, and murine typhus. No epidemic typhus has been identified in Taiwan since World War II. Murine typhus, caused by *Rickettsia typhi*, is endemic to Taiwan, with approximately 20–60 cases each year. Scrub typhus group rickettsiosis is the most prevalent infection and causes 300–500 confirmed cases annually (http://www.cdc.gov.tw/). Scrub typhus is endemic to Asia, northern Australia, and the western Pacific regions [1, 2]. Diverse strains of *Orientia tsutsugamushi*, the etiological agent of scrub typhus, have been isolated from humans and its vectors, larval-stage trombiculid mites (chiggers) [3, 4]. Epidemiological surveys have suggested considerably high prevalence of *O. tsutsugamushi* infections in wild rodents, ranging from 69.1 % to over 90 %, on the offshore and main islands of Taiwan [5, 6]. Human infections of scrub typhus are routinely diagnosed using molecular, serological and pathogen isolation with shell-vial methods performed at the Centers for Disease Control in Taiwan (Taiwan CDC).

*Ehrlichia* spp., another genus of bacteria responsible for rickettsial diseases in humans and wild or domestic animals, is less recognized in Taiwan. The first human case of ehrlichiosis was documented in the United States of America in 1986. Ehrlichiosis has since been reported worldwide [7]. The common pathogens that cause human infections are *Ehrlichia chaffeensis* and *E. ewingii*. The symptoms are moderate to severe and occasionally are fatal [8, 9]. Enzootic cycles of *Ehrlichia* spp. transmitted between ticks and wild animals are widespread and have been reported in Korea, Japan, and southern China, which are in geographic proximity to Taiwan [10, 11]. Previous studies have identified *E. chaffeensis* in *Rhipicephalus haemaphysaloides* and *Ixodes granulatus* ticks and rodents in Kinmen, one of Taiwan’s offshore islands [12]. Here, we report ehrlichial infections in small mammal reservoirs on the main island. The potential impact of human ehrlichiosis in Taiwan has yet to be determined.

The impact of travel on the spread of infectious diseases is of great concern. Human migration has provided a shortcut for disease dissemination. When people travel, they often carry luggage, food and goods, and they are also accompanied by, as their biological microenvironments, microbes, animals, and parasites. To examine the risks arising from transportation of pathogenic *Orientia* and *Ehrlichia* through human activities, sera were collected from small mammals captured around harbors, and the prevalence of seropositivity against *O. tsutsugamushi* and *Ehrlichia* spp. was determined by immunofluorescence assay (IFA). To the best of our knowledge, this is the first systematic surveillance of ehrlichial and *Orientia tsutsugamushi* infections in their reservoirs on the main island of Taiwan.

## Methods

### Study area and small mammal capture

**Fig. 1:**
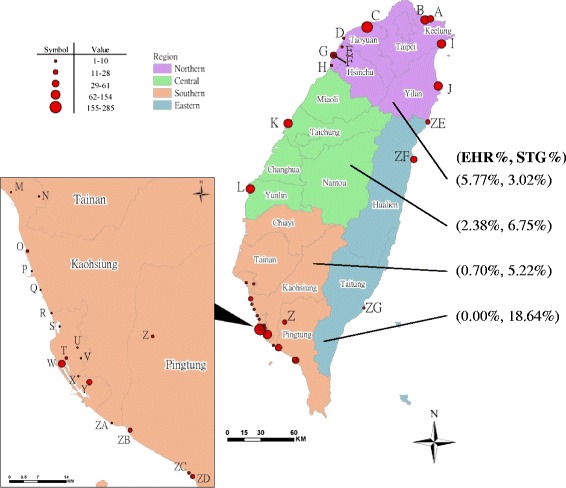
Thirty-three locations where small mammals were captured around Taiwan. (Purple: northern Taiwan; green: central Taiwan; orange: southern Taiwan; and blue: eastern Taiwan. Value is number of small mammals captured at each site.)

This study was conducted as part of the surveillance of the Hanta virus in rodents in Taiwan [13]. Small mammals were captured from 33 study sites, including 8 international harbors, 18 domestic fishing harbors, and 7 local public health centers around Taiwan from November 2004 to December 2008 (Fig. 1 and Table 1). The methods for small mammal capture have been described in a previous report [14]. Briefly, approximately 20–30 traps were set up each month in areas with suspected rodent activities. Trapping at international ports took place for three days each month. Sweet potatoes, peanuts and/or sausages were used as bait to attract diverse species of small mammals. Blood was collected by cardiac puncture after the captured animals were anesthetized with Zoletil 50 (Fa. Virbac, Carros, France) and was left to stand for 1 h at room temperature. Sera were separated by centrifugation, aliquoted, and kept frozen at −20 °C for later analysis.

**Table 1:** List of the 33 locations around Taiwan where small mammals were captured and rates of seropositivity against *Ehrlichia* spp. and *Orientia tsutsugamushi* from November 2004 to December 2008

Geographic	Location (^a^)	Total no. trapped mammals (No. sero-positive to EHR, no. sero-positive to STG)					
		2004	2005	2006	2007	2008	Total (2004 ~ 2008)
Northern		0	140 (2,2)	186 (16,6)	134 (12,4)	302 (14,11)	762 (44,23)
	A. Badouzih Fishing Harbor (KL)	0	33	25	0	0	58
	B. Keelung Harbor (KL)^b^	0	44 (2,2)	38 (3,2)	22 (0,1)	30 (1,2)	134 (6,7)
	C. Taoyuan Airport (TY)^b^	0	30	71 (8,2)	80 (12,1)	64 (2,1)	245 (22,4)
	D. Yongan Fishing Harbor (TY)	0	0	0	0	6	6
	E. Potou Fishing Harbor (HC)	0	0	1	0	0	1
	F. Nangliao Fishing Harbor (HC)	0	0	9	0	0	9
	G. Hsinchu Fishing Harbor (HC)	0	0	0	0	61 (2,2)	61 (2,2)
	H. Haishan Fishing Harbor (HC)	0	0	0	0	5	5
	I. Dashi Fishing Harbor (YL)	0	0	5	0	84 (6,3)	89 (6,3)
	J. Suao Harbor (YL)^b^	0	33	37 (5,2)	32 (0,2)	52 (3,3)	154 (8,7)
Central		1	100 (2,6)	66 (4,9)	40	45 (0,2)	252 (6,17)
	K. Taichung Wuchi Harbor (TC)^b^	0	27	34	22	29	112
			(0,1)	(1,2)	(0,0)	(0,2)	(1,5)
	L. Mailiao Harbor (YL)^b^	1	73 (2,5)	32 (3,7)	18 (0,0)	16 (0,0)	140 (5,12)
Southern		10	193 (0,4)	130 (3,4)	85 (0,10)	157 (1,12)	575 (4,30)
	M. Anping Fishing Harbor (TN)	0	0	2	0	0	2
	N. East District, Tainan city (TN)	0	0	2	0	0	2
	O. Hsinda Fishing Harbor (KH)	0	0	16	3	9	28
	P. Hsingang Fishing Harbor (KH)	0	0	1	0	0	1
	Q. Mito Fishing Harbor (KH)	0	0	0	0	4	4
	R. Uhliao Fishing Harbor (KH)	0	0	2	3	5	10
	S. Zhoying Military Area (KH)	0	0	7	0	0	7
	T. Qianzhen District, Kaohsiung City (KH)	0	0	0	0	15	15
	U. Sanmin District, Kaohsiung City (KH)	0	0	0	0	1	1
	V. Yanchao District, Kaohsiung City (KH)	0	0	4	0	0	4
	W. Kaohsiung International Harbor (KH)^b^	8	65 (0,2)	51 (0,1)	67 (0,7)	94 (1,9)	285 (1,19)
	X. Chungyun Fishing Harbor (KH)	0	0	3	0	0	3
	Y. Kaohsiung International Airport (KH)^b^	2	41	30 (2,3)	12 (0,3)	11 (0,0)	96 (2,6)
	Z. Yanpu Fishing Harbor (PT)	0	0	0	0	16 (0,3)	16 (0,3)
	ZA. Linyuan Fishing Harbor (KH)	0	0	0	0	2	2
	ZB. Donggang Fishing Harbor (PT)	0	32	6	0	0	38
	ZC. Shueidiliao Fishing Harbor (PT)	0	16	0	0	0	16
	ZD. Fangliao Fishing Harbor (PT)	0	39 (0,2)	6 (1,0)	0	0	45 (1,2)
Eastern		0	6 (0,1)	9 (0,1)	19 (0,2)	25 (0,7)	59 (0,11)
	ZE. Hoping Fishing Harbor (HL)	0	0	0	4 (0,2)	16 (0,6)	20 (0,8)
	ZF. Hualien Harbor (HL)^b^	0	6 (0,1)	6 (0,1)	15	9 (0,1)	36 (0,3)
	ZG. Fugang Fishing Harbor (TT)	0	0	3	0	0	3
Total		11 (0,0)	439 (4,13)	391 (23,20)	278 (12,16)	529 (15,32)	1648 (54,81)

*KL* Keelung, *TY* Taoyuan, *HC* Hsinhu, *TC* Taichung, *YL* Yunlin, *TN* Tainan, *KH* Kaohsiung, *PT* Pingtung, *TT* Taitung, *HL* Hualien, *YL* Yilan

^a^Name of County or City in Taiwan

^b^International Harbor

### Ethical considerations

The study was conducted following the regulations of Article 17 of Taiwan Wildlife Conservation Act and with the permission of Taiwan Centers for Disease Control. None of the trapped mammals belonged to Protected Species. All trapping and sampling procedures were performed according to U.S. CDC guidelines for sampling small mammals for virologic testing and met International Health Regulations (IHR2005). The use of animal sera for antibody detection was approved by National Taiwan University College of Medicine and College of Public Health Institutional Animal Care and Use Committee (IACUC Approval No: 20090296).

### Serological analysis

Rodent serum samples were evaluated for reactivity to *E. chaffeensis* (prepared by Prof. Yu’s laboratory, the University of Texas Medical Branch) and *O. tsutsugamushi* (Karp and Kato strains; prepared by Taiwan CDC) using IFA.

For detecting antibodies specific to *Ehrlichia* spp., antigen slides were prepared with *E. chaffeensis* Arkansas-infected DH82 cells. Briefly, *E. chaffeensis*-infected DH82 cells from a 150-cm^2^ flask were collected when 100 % of the cells were infected. The cells were centrifuged at 200 × g for 10 min. Pellets were resuspended in 10 ml of phosphate-buffered saline (PBS) with 0.1 % bovine albumin. Sodium azide was added to a final concentration of 0.01 % and treated at 4 °C overnight to inactivate *Ehrlichia*. Ten microliters of antigen was applied to each well of 12-well slides. The slides were air dried and fixed in acetone for 5 min before immunostaining [15].

For the detection of scrub typhus antibodies, *O. tsutsugamushi* (Karp strain)-infected L929 cells on chamber slides were fixed and permeabilized with ice-cold acetone/methanol (1:1) at −20 °C for 10 min. The slides were dried and blocked with PBS containing 1 % goat serum before further immunostaining [16].

Serum samples were centrifuged at 1730 × g at 4 °C for 10 min and diluted to 1:80 using a sterile field diluent (PBS containing 1.5 % bovine albumin, 100 units/mL of penicillin, 100 μg/mL of streptomycin, and 250 μg/mL of amphotericin B). A 20 μL volume of rodent serum was pipetted into each well, and the slides were incubated in a moist chamber at 37 °C for 30 min. The slides were rinsed and washed in PBS for 10 min and then rinsed again with distilled water. The slides were air-dried, and the wells were incubated with 20 μL of fluoresce in isothiocyanate (FITC)-conjugated, affinity-purified, Fc fragment-specific, goat anti-rat IgG (Jackson Immuno-Research Laboratories, West Grove, PA) diluted to 1:100 in PBS containing Evans blue counterstain (Sigma Chemical Company, St. Louis, MO). After incubation in a moist chamber at 37 °C for 30 min, the slides were rinsed and washed with PBS twice. Coverslips were mounted using mounting medium (PBS:glycerol = 3:7 v/v; pH 9.0), and the slides were examined at a magnification of 100× or 400× with a Zeiss fluorescent microscope. Sera with typical patterns of bright green fluorescence at titers of 1:80 for IgG were deemed positive [17, 18]. Positive and negative control sera were included on each slide. All slides were read independently by two individuals who were blinded to specimen identity. Disagreements were resolved by consensus after re-examining the slides.

### Statistical analysis

All statistical analyses were performed using SAS v9.1.3 software (SAS Institute, Cary, NC). Fisher’s exact test was used for comparisons of seroprevalence in rodents between different sampling sites. A two-tailed *p*-value of 0.05 or less was considered to be statistically significant. The trend across time was examined with the Cochran-Mantel-Haenszel Chi-squared test.

## Results

### Field collection of small mammals

A total of 1648 small mammals were caught from 33 study sites in Taiwan from November 2004 to December 2008, including 762, 252, 575 and 59 animals in northern, central, southern, and eastern Taiwan, respectively (Table 1). Figure 1 summarizes the numbers of trapped small mammals at the different sites. The seroprevalence of ehrlichial and *O. tsutsugamushi* infections in 4 districts of Taiwan was determined. Six species of rodents, *Rattus norvegicus* (brown rat), *Bandicota indica* (Taiwan bandicoot rat), *R. losea* (brown country rat), *Mus musculus* (house mouse), *R. tanezumi* (oriental house rat), and *Apodemus agrarius* (striped field mouse), and a species of insectivore, *Suncus murinus* (Asian house shrew), were identified in this study. The numbers of each species captured and the animals’ genders are outlined in Table 2. *Rattus norvegicus*, *S. murinus*, and *B. indica* were the predominant species, accounting for 1102, 284 and 182 of the trapped small mammals, respectively.

**Table 2:** Species and genders of the captured small mammals and the prevalence of antibodies against *Ehrlichia* spp. and *Orientia tsutsugamushi* in small mammals in Taiwan from November 2004 to December 2008

			EHR		STG		*p* value
Species	Sex	Total	Positive	%	Positive	%	
*Rattus norvegicus*	M	602	17	2.82	36	5.98	0.0293
	F	489	13	2.66	31	6.34	0.0244
	U	11	3	27.27	2	18.18	1.0000
	total	1102	33	2.99	69	6.26	0.0023
*Suncus murinus*	M	102	0	0.00	4	3.92	0.1213
	F	179	0	0.00	0	0.00	-
	U	3	0	0.00	0	0.00	-
	total	284	0	0.00	4	1.41	0.1237
*Bandicota indica*	M	130	13	10.00	1	0.77	0.0014
	F	52	6	11.54	1	1.92	0.1123
	total	182	19	10.44	2	1.10	0.1886
*Rattus losea*	M	35	2	5.71	1	2.86	1.0000
	F	15	0	0.00	2	13.33	0.4828
	total	50	2	4.00	3	6.00	1.0000
*Mus musculus*	M	6	0	0.00	0	0.00	-
	F	10	0	0.00	0	0.00	-
	total	16	0	0.00	0	0.00	-
*Rattus tanezumi*	M	5	0	0.00	0	0.00	-
	F	8	0	0.00	3	37.50	0.2000
	total	13	0	0.00	3	23.08	0.2200
*Apodemus agrarius*	M	1	0	0.00	0	0.00	-
	F	0	0	-	0	--	-
	total	1	0	0.00	0	0.00	-
Total		1648	54	3.28	81	4.92	

*M* male, *F* female, *U* unknown

### Serological analyses of captured animals

Antibodies against *Ehrlichia* spp. and *O. tsutsugamushi* were found in 3.28 % (54/1648) and 4.92 % (81/1648) of the captured small mammals on the main island of Taiwan, respectively (Table 2). The prevalence was significantly different between ehrlichial (2.99 %; 33/1102) and *O. tsutsugamushi* infections (6.26 %; 69/1102) in *R. norvegicus* (*p* < 0.01). The gender of the animals captured appeared to have no influence on the prevalence of both rickettsial infections. Among the rodents, *B. indica* displayed the highest positive rate of 10.44 % (19/182) against *Ehrlichia* spp., and *Ratus tanezumi* exhibited the highest infection rate against *O. tsutsugamushi* (23.08 %; 3/13).

### Seroprevalence in small mammals captured in different geographic locations in Taiwan

The serological survey revealed that antibodies specific to *Ehrlichia* spp. were most frequently found in small mammals active around the ports in northern Taiwan (5.77 %; 44/762), followed by the central (2.38 %; 6/252) and southern (0.70 %; 4/575) districts. The percentage of samples positive for antibodies specific to *Ehrlichia* spp. was highest in Taoyuan Airport (8.98 %; 22/245). Furthermore, in agreement with the incidence in human cases, we detected a high prevalence of antibodies against *O. tsutsugamushi* at the Hoping Fishing Harbor (40 %; 8/20), which is located in eastern Taiwan (Table 1 and Fig. 1).

In this study, 1202 and 446 small mammals were trapped at the international and domestic harbors (including local public health centers), respectively (Table 3). The prevalence of antibodies specific to *O. tsutsugamushi* was slightly higher than the prevalence of antibodies specific to *Ehrlichia* spp. Moreover, a higher but not significantly greater number of animals expressed antibodies against the rickettsial diseases around the international ports. Most importantly, serological evidence of infections was detected in all international harbors (Table 1).

**Table 3:** Comparison of rates of seropositivity against *Ehrlichia* spp. (EHR), and *Orientia tsutsugamushi* (STG) infections between international harbors and local harbors in Taiwan

		EHR		STG	
Locations	Total	Positive	%	Positive	%
International Harbor	1202	45	3.74	63	5.24
Local Harbor	446	9	2.02	18	4.04
Total	1648	54	3.28	81	4.92

### Seroprevalence in small mammals captured at different time points

**Fig. 2:**
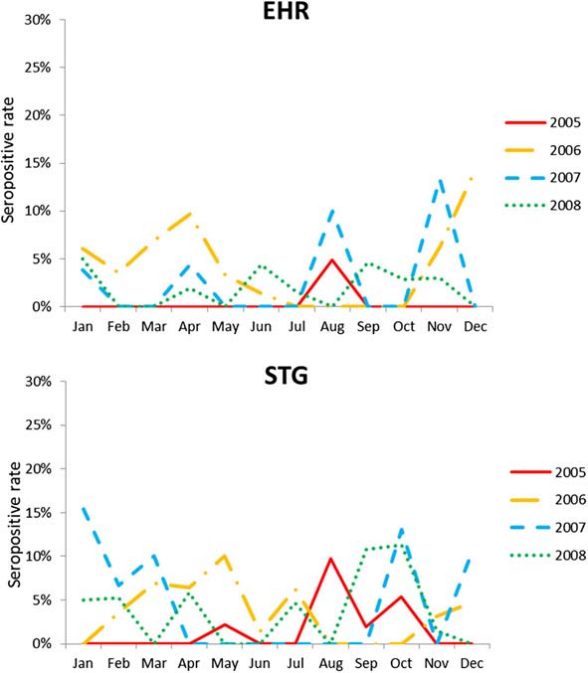
Seasonal prevalence of antibodies against *Ehrlichia* spp. (EHR), and *Orientia tsutsugamushi* (STG) in small mammals from November 2004 to December 2008 in Taiwan

The seroprevalence of *O. tsutsugamushi* infections in small mammals increased in the harbors of southern Taiwan during the year of 2004 and 2008 (*p* < 0.01) (Table 4). Antibodies against *Ehrlichia* spp. and *O. tsutsugamushi* were detected year-round (Fig. 2). In trapped small mammals, the seropositive rates were highest in November and December for *Ehrlichia* spp., whereas the positive rates were higher in January and October for *O. tsutsugamushi*.

**Table 4:** Trends of rates of seropositivity of antibodies against *Ehrlichia* spp. (EHR) and *Orientia tsutsugamushi* (STG) in small mammals in northern, central, southern, and eastern Taiwan from November 2004 to December 2008

	2004 (*N* = 11)		2005 (*N* = 439)		2006 (*N* = 391)		2007 (*N* = 278)		2008 (*N* = 529)			(trend)
Variable	N	%	N	%	N	%	N	%	N	%	*p* value	*p* value
EHR												
Northern	0	0	2	0.46	16	2.81	12	4.32	14	0.95	0.0054	0.8555
Central	0	0	2	0.46	4	1.02	0	0.00	0	0.00	0.1908	0.3183
Southern	0	0	0	0.00	3	0.77	0	0.00	1	0.19	0.0845	0.8228
Eastern	0	0	0	0.00	0	0.00	0	0.00	0	0.00	-	-
Total	0	0	4	0.91	23	5.88	12	4.32	15	2.84	0.0003	0.3306
*p* value			0.127		0.115		0.00007375		0.040			
STG												
Northern	0	0	2	0.46	6	1.02	4	0.72	11	0.95	0.9432	0.6543
Central	0	0	6	1.37	9	2.30	0	0.00	2	0.38	0.0440	0.3680
Southern	0	0	4	0.91	4	0.77	10	3.60	12	2.27	0.0016	0.0023
Eastern	0	0	1	0.23	1	0.77	2	1.44	7	2.46	0.7194	0.2352
Total	0	0	13	2.96	20	4.86	16	5.76	32	6.05	0.1183	0.0264
* p* value			0.245		0.005		0.008		0.167

### Co-infection with different pathogens in captured animals

Of the 1648 small mammals tested in the study, 5 (0.30 %) were seropositive against both *Ehrlichia* spp. and *O. tsutsugamushi*, including one *R. norvegicus* in Keelung Harbor, 2 *R. norvegicus* in Mailiao Harbor, one *M. musculus* in Kaohsiung International Airport, and one *R. norvegicus* in Suao Harbor.

## Discussion

In the study, the prevalence of *Ehrlichia* spp. and *O. tsutsugamishi* infections in captured animals was 3.28 % (54/1648) and 4.92 % (81/1648), respectively. Most of the seropositive animals were located in northern Taiwan. Serological evidence of infections were present in all international ports. The antibodies were detected in trapped small mammals year-round with an increasing trend in *O. tsutsugamushi* infections in southern Taiwan from 2004 to 2007. Moreover, this is the first report regarding the prevalence of *Ehrlichia* spp. infection in small mammals on the main island of Taiwan.

Ehrlichiosis is an important emerging zoonotic disease. Serological studies and molecular detections have associated *E. chaffeensis* with white-tailed deer, goats, red foxes, coyotes, raccoons, cattle, and Reeves’s muntjacs [7, 19–25]. These large wild animals are less common in Taiwan. Conversely, shrews and rodents are more abundant and often serve as reservoirs for many communicable diseases transmitted by their ectoparasites. Previous studies have discovered *E. chaffeensis* DNA in 16 of 108 (14.8 %) spleen or liver samples from shrews and rodents on one of Taiwan’s offshore islands, Kinmen, by polymerase chain reaction (PCR) and sequencing [14]. Our results further showed that 3.28 % (54/1648) of captured animals were seropositive against ehrlichial infections, and more infected animals were found in the north and central parts of the main island of Taiwan. During this study, ectoparasites collected from murine-like animals included two fleas: *X. cheopis* and *Nosopsyllus nicanus*, three ticks: *Ixodes granulatus*, *Haemaphysalis bandicota,* and *Rhipicephalus haemaphysaloides*, three mites: *Laelaps nuttalli*, *L. sedlaceki*, and *L. echidninus*, and lice. Among these ectoparasites, 45.04 % were *X. cheopis*, and 29.25 % were *H. bandicota*. In terms of the ectoparasites, *X. cheopis* was the most common species. *X. cheopis* carries *R. typhi* and serves as a major vector to cause human murine typhus. *Haemaphysalis bandicota*, the second common ectoparasite, involves in disease transmission among rats. However, *H. bandicota* has never been found to be associated with any vector-borne communicable diseases in humans [26]. Information on trombiculid mites was limited in this study, but these mites may be associated with small mammal species. It is worth noting that serological cross-activities have been addressed among *Ehrlichia* spp., such as *E. chaffeensis*, *E. ewingii*, *E. canis* and *E. ruminantium* [27–30]. The enzootic cycle of *E. canis* has been documented in dogs in Taiwan [31, 32]. The positive reactions observed by IFA in the current study therefore may have been confounded by infections with other *Ehrlichia* species. In North America, the seroprevalence of *E. canis*, *E. chaffeensis* and *E. ewingii* in dogs was 0.8 %, 2.8 %, and 5.1 %, respectively [33]. In China, the overall seroprevalence of *E. chaffeensis* was 9.8 % in rural residents and 2.4 % in urban residents [34]. Nevertheless, our results suggested that ehrlichial infections are prevalent on the island. The implications for human ehrlichiosis should be scrutinized and re-evaluated because it is not a commonly observed disease in Taiwan.

Several studies have evaluated the prevalence of scrub typhus infections in animal reservoirs. A seropositive rate of over 90 % has been reported in rodents in Kinmen [5]. A prevalence of 70 % has been observed in small mammals captured in abandoned agricultural fields in central Hualien County in eastern Taiwan [35]. Our results showed that 4.92 % of the small mammals caught around the harbors expressed antibodies against *O. tsutsugamushi*. The prevalence was relatively low compared with those in previous reports and may reflect the different ecosystems of our sampling areas. Different rodent hosts affect the seropositive rates of rickettsial infections [5, 12, 13]. Our study focused on small mammals active around harbors, which have, to a certain degree, much more human activities than present in the study sites selected in other studies. Furthermore, the rodent species in these harbors differed from those on small islets or in traditional agricultural environments. Indeed, the dominant species among captured animals varies in the literature. *R. norvegicus* was the dominant rodent species in this study, whereas *R. losea* and *Apodemus agrarius* are abundant in Kinmen and Hualien, respectively [5, 35]. The distributions of the rodent species were similar among harbors in Taiwan, with the exceptions of Suao Harbor and Taoyuan Airport. On the whole, *R. norvegicus* was the most frequent species followed by *S. murinus* and *B. indica*. However, the Taiwan bandicoot rat and brown country rat were the primary rodents captured in Taoyuan Airport. This may be related to the trap sites and the behaviors of *B. indica*, which are usually active in non-residential areas. *Haemaphysalis bandicota* was also primarily found at Taoyuan International Airport [26]. For Su-ao Harbor, the only species captured were *R. norvegicus* and *M. musculus* (*n* = 1).

Together, the geographic distribution, activities of the vectors and reservoirs, and human activities determine the prevalence of rickettsial disease infections. In this study, *B. indica* appeared to be most influenced by *E. chaffeensis* (10.44 %), and *R. tanezumi* had the highest rate of seropositivity against *O. tsutsugamushi* (23.08 %). Although seropositive rates were highest in November and December for *Ehrlichia* spp., whereas the positive rates were higher in January and October for *O. tsutsugamushi*. The numbers of captured animals did not differ significantly between seasons, and the antibodies were detected year-round. In general, our results revealed that small mammals, which are common in residences, were infected by *Ehrlichia* and *O. tsutsugamushi*. The frequent association between these mammals and human activities may increase the risk of rickettsial diseases.

Our results indicated that the rates of seropositivity against *O. tsutsugamushi* were higher in the eastern and central parts of Taiwan, which correlated with the high incidence of human scrub typhus in these regions [3]. Multiple environmental factors and vector species have been shown to affect the prevalence of vector-borne infectious pathogens in animal hosts [36–38]. A recent article has also reported a positive correlation between the rate of seropositivity in rodents and the incidence of human scrub typhus; however, the major captured animal in that study, *S. murinus*, was not tested due to a lack of antiserum [38]. FITC-conjugated goat anti-rat antibodies were used to detect antibodies from captured small mammals in our study. Similar strategies have been applied in other experiments when no antiserum was commercially available [39, 40]. Although we cannot guarantee that anti-rat secondary antibodies react with rodent and shrew sera equally, the shrew was a minor species among all trapped small mammals (17.23 %). Zero and 4 (1.41 %) shrews tested positive for antibodies against *E. chaffeensis* and *O. tsutsugamushi*, respectively.

Travel and trade have provided a source for disease dissemination throughout recorded history. Plague represents one of the most striking examples of this phenomenon. Human population movement has been crucial to the dispersal of *Yersinia pestis* and rodents and their fleas to new territories [41]. Moreover, international trade of pet reptiles and amphibians has been shown to have a potential to cause emerging infections of *Rickettsia* and *Ehrlichia* spp. in Japan [42]. In our study, animals were found to harbor antibodies against *Ehrlichia* and *O. tsutsugamushi* around ports and harbors. The prevalence of seropositivity was slightly higher in the samples collected near international harbors; however, no significant differences were observed. Nevertheless, serological evidence of infections was detected in all international harbors studied. These findings highlight the risk of spreading pathogenic *Ehrlichia* and *Orientia* associated with ectoparasites on rodents through aerial and marine transportation.

## Conclusions

Our study provided serological evidence of *Ehrlichia* spp. infection in small mammals on the main island of Taiwan. The implications for human ehrlichiosis should be re-evaluated because it is not a commonly observed disease in Taiwan. Small mammals can serve as useful indicators and/or sentinels for epidemiological surveillance, disease control and prevention of vector-borne infectious diseases. The prevalence of infected animals captured around the harbors should raise awareness of the potential risks of disseminating pathogens by domestic and international aerial and marine transport.

## Abbreviations

IFA: immunofluorescence assay
IgG: immunoglobulin G
PBS: phosphate-buffered saline

## References

[1] Tamura A, Ohashi N, Urakami H, Miyamura S (1995). Classification of *Rickettsia tsutsugamushi* in a new genus, *Orientia* gen. nov., as *Orientia tsutsugamushi* comb. nov. Int J Syst Bacteriol.

[2] Walker DH (2003). Rickettsial diseases in travelers. Travel Med Infect Dis.

[3] Lu HY, Tsai KH, Yu SK, Cheng CH, Yang JS, Su CL (2010). Phylogenetic analysis of 56-kDa type-specific antigen gene of *Orientia tsutsugamushi* isolates in Taiwan. Am J Trop Med Hyg.

[4] Lin PR, Tsai HP, Tsui PY, Weng MH, Kuo MD, Lin HC (2011). Genetic typing, based on the 56-kilodalton type-specific antigen gene, of *Orientia tsutsugamushi* strains isolated from chiggers collected from wild-caught rodents in Taiwan. Appl Environ Microbiol.

[5] Wang HC, Chung CL, Lin TH, Wang CH, Wu WJ (2004). Studies on the vectors and pathogens of scrub typhus on murine-like animals in Kinmen County, Taiwan. Formosa Entomol.

[6] Lin PR, Tsai HP, Weng MH, Lin HC, Chen KC, Kuo MD (2014). Field assessment of *Orientia tsutsugamushi* infection in small mammals and its association with the occurrence of human scrub typhus in Taiwan. Acta Trop.

[7] Yabsley MJ (2010). Natural history of *Ehrlichia chaffeensis*: vertebrate hosts and tick vectors from the United States and evidence for endemic transmission in other countries. Vet Parasitol.

[8] Dumler JS, Madigan JE, Pusterla N, Bakken JS (2007). Ehrlichioses in humans: epidemiology, clinical presentation, diagnosis, and treatment. Clin Infect Dis.

[9] Dumler JS, Barbet AF, Bekker CP, Dasch GA, Palmer GH, Ray SC (2001). Reorganization of genera in the families Rickettsiaceae and Anaplasmataceae in the order Rickettsiales: unification of some species of *Ehrlichia* with *Anaplasma*, *Cowdria* with *Ehrlichia* and *Ehrlichia* with *Neorickettsia*, descriptions of six new species combinations and designation of *Ehrlichia equi* and ‘HGE agent’ as subjective synonyms of *Ehrlichia phagocytophila*. Int J Syst Evol Microbiol.

[10] Cao WC, Gao YM, Zhang PH, Zhang XT, Dai QH, Dumler JS (2000). Identification of *Ehrlichia chaffeensis* by nested PCR in ticks from Southern China. J Clin Microbiol.

[11] Li HM, Jiang BG, He J, Niu JJ, Wang JX, Sun Y (2006). Detection and identification of *Ehrlichia* sp. in *Boophilus microplus* ticks from Xiamen of Fujian Province. J Patho Biol.

[12] Weng MH, Lien JC, Tsai HP, Lin PR, Guo MD, Liu WT (2010). *Ehrlichia chaffeensis* infection in rodent ticks - Kinmen, 2009. Taiwan. Taiwan Epi Bul.

[13] Hsieh JW, Wang JT, Hiuang TM, Chen CH (2008). Epidemiology investigation of rodents as vectors for the hantavirus in Taiwan’s harbor area. Taiwan Epi Bull.

[14] Weng MH, Tsai HP, Lin PR, Cheng KC, Guo MD, Liu WT (2014). Investigation of *Ehrlichia chaffeensis* infections in rodents in Kinmen Area, 2012. Taiwan Epi Bull.

[15] Yu XJ, Crocquet-Valdes PA, Cullman LC, Popov VL, Walker DH (1999). Comparison of *Ehrlichia chaffeensis* recombinant proteins for serologic diagnosis of human monocytotropic ehrlichiosis. J Clin Microbiol.

[16] Tsai KH, Wang HC, Chen CH, Huang JH, Lu HY, Su CL (2008). Isolation and identification of a novel spotted fever group rickettsia, strain IG-1, from *Ixodes granulatus* ticks collected on Orchid Island (Lanyu), Taiwan. Am J Trop Med Hyg.

[17] Magnarelli LA, Anderson JF, Stafford KC, Dumler JS (1997). Antibodies to multiple tick-borne pathogens of babesiosis, ehrlichiosis, and Lyme borreliosis in white-footed mice. J Wildl Dis.

[18] Marshall GS, Jacobs RF, Schutze GE, Paxton H, Buckingham SC, DeVincenzo JP (2002). *Ehrlichia chaffeensis* seroprevalence among children in the southeast and south-central regions of the United States. Arch Pediatr Adolesc Med.

[19] Paddock CD, Childs JE (2003). *Ehrlichia chaffeensis*: a prototypical emerging pathogen. Clin Microbiol Rev.

[20] Dugan VG, Little SE, Stallknecht DE, Beall AD (2000). Natural infection of domestic goats with *Ehrlichia chaffeensis*. J Clin Microbiol.

[21] Dugan VG, Gaydos JK, Stallknecht DE, Little SE, Beall AD, Mead DG (2005). Detection of *Ehrlichia* spp. in raccoons (*Procyon lotor*) from Georgia. Vector Borne Zoonotic Dis.

[22] Kocan AA, Levesque GC, Whitworth LC, Murphy GL, Ewing SA, Barker RW (2000). Naturally occurring *Ehrlichia chaffeensis* infection in coyotes from Oklahoma. Emerg Infect Dis.

[23] Davidson WR, Lockhart JM, Stallknecht DE, Howerth EW (1999). Susceptibility of red and gray foxes to infection by *Ehrlichia chaffeensis*. J Wildl Dis.

[24] Wen B, Cao W, Pan H (2003). *Ehrlichiae* and ehrlichial diseases in China. Ann NY Acad Sci.

[25] Lockhart JM, Davidson WR, Stallknecht DE, Dawson JE, Little SE (1997). Natural history of *Ehrlichia chaffeensis* (Rickettsiales: Ehrlichieae) in the piedmont physiographic province of Georgia. J Parasitol.

[26] Chien CH, Chiang PF, Wang HC, Chen KY, Lin MC, Wu HS (2012). Prevalence of ectoparasites and the seroepidemiology of murine typhus in murine-like animals at international ports in Taiwan, 2004–2011. Taiwan Epidemiol Bull.

[27] Maeda K, Markowitz N, Hawley RC, Ristic M, Cox D, McDade JE (1987). Human infection with *Ehrlichia canis*, a leukocytic rickettsia. N Engl J Med.

[28] Kelly PJ, Matthewman LA, Mahan SM, Semu S, Peter T, Mason PR (1994). Serological evidence for antigenic relationships between *Ehrlichia canis* and *Cowdria ruminantium*. Res Vet Sci.

[29] Buller RS, Arens M, Hmiel SP, Paddock CD, Sumner JW, Rikhisa Y (1999). *Ehrlichia ewingii,* a newly recognized agent of human ehrlichiosis. N Engl J Med.

[30] Jongejan F, de Vries N, Nieuwenhuijs J, Van Vliet AH, Wassink LA (1993). The immunodominant 32-kilodalton protein of *Cowdria ruminantium* is conserved within the genus *Ehrlichia*. Rev Elev Med Vet Pays Trop.

[31] Huang CC, Hsieh YC, Tsang CL, Chung YT (2010). Sequence and phylogenetic analysis of the gp200 protein of *Ehrlichia canis* from dogs in Taiwan. J Vet Sci.

[32] Yuasa Y, Hsu TH, Chou CC, Huang CC, Huang WC, Chang CC (2012). The comparison of spatial variation and risk factors between mosquito-borne and tick-borne diseases: Seroepidemiology of *Ehrlichia canis*, *Anaplasma* species, and *Dirofilaria immitis* in dogs. Comp Immunol Microbiol Infect Dis.

[33] Beall MJ, Alleman AR, Breitschwerdt EB, Cohn LA, Couto CG, Dryden MW (2012). Seroprevalence of *Ehrlichia canis*, *Ehrlichia chaffeensis* and *Ehrlichia ewingii* in dogs in North America. Parasit Vectors.

[34] Zhang L, Liu H, Xu B, Zhang Z, Jin Y, Li W (2014). Rural residents in China are at increased risk of exposure to tick-borne pathogens *Anaplasma phagocytophilum* and *Ehrlichia chaffeensis*. BioMed Res Int.

[35] Kuo CC, Huang CL, Wang HC (2011). Identification of potential hosts and vectors of scrub typhus and tick-borne spotted fever group rickettsiae in eastern Taiwan. Med Vet Entomol.

[36] Cooper WC, Lien JC, Hsu SH, Chen WF (1964). Scrub typhus in the Pescadores Islands: An epidemiologic and clinical study. Am J Trop Med Hyg.

[37] Lee YS, Wang PH, Tseng SJ, Ko CF, Teng HJ (2006). Epidemiology of scrub typhus in eastern Taiwan, 2000–2004. Jpn J Infect Dis.

[38] Kuo CC, Huang JL, Ko CY, Lee PF, Wang HC (2011). Spatial analysis of scrub typhus infection and its association with environmental and socioeconomic factors in Taiwan. Acta Tropica.

[39] Luan VD, Yoshimatsu K, Endo R, Taruishi M, Huong VT, Dat DT (2012). Studies on hantavirus infection in small mammals captured in southern and central highland area of Vietnam. J Vet Med Sci.

[40] Guan D, Li W, Su J, Fang L, Takeda N, Wakita T (2013). Asian musk shrew as a reservoir of rat hepatitis E virus, China. Emerg Infect Dis.

[41] Wilson ME (1995). Travel and the emergence of infectious diseases. Emerg Infect Dis.

[42] Andoh M, Sakata A, Takano A, Kawabata H, Fujita H, Une Y (2015). Detection of *Rickettsia* and *Ehrlichia* spp. In ticks associated with exotic reptiles and amphibians imported into Japan. PLoS ONE.

